# Oral manifestations in vitamin B_12_ deficiency patients with or without history of gastrectomy

**DOI:** 10.1186/s12903-016-0215-y

**Published:** 2016-05-27

**Authors:** Jihoon Kim, Moon-Jong Kim, Hong-Seop Kho

**Affiliations:** Department of Oral Medicine and Oral Diagnosis, School of Dentistry and Dental Research Institute, Seoul National University, Yunkeun-Dong 28, Chongro-Ku, Seoul 110-749 Republic of Korea; Institute on Aging, Seoul National University, Gwanak-Ro 1, Gwanak-Gu, Seoul 151-742 Republic of Korea

**Keywords:** Oral, Vitamin B_12_, Gastrectomy

## Abstract

**Background:**

The purpose of this study was to compare clinical features of vitamin B_12_ deficiency patients with a history of gastrectomy to those without a history of gastrectomy.

**Methods:**

Twenty-two patients with vitamin B_12_ deficiency were included. Patients’ chief complaints, oral manifestations, blood examination results, and past medical histories were reviewed.

**Results:**

Eleven patients had a history of gastrectomy and 11 did not. The chief complaint was glossodynia in all patients. No significant differences were observed between the two groups regarding age, sex, symptom duration, or plasma vitamin B_12_ level. Erythema and depapillation of the tongue were the most common findings, however less common among patients without a history of gastrectomy. Two patients with a history of gastrectomy and 5 patients without a history of gastrectomy had normal oral mucosa. Patients with a history of gastrectomy were more anemic. Oral symptoms of the majority of patients responded to antifungals and vitamin B_12_ replacement. The suggested etiologies for vitamin B_12_ deficiency in the patients without a history of gastrectomy were gastritis, medications, diet, autoimmunity, and early gastric cancer.

**Conclusions:**

Vitamin B_12_ deficiency and its associated etiological factors should be considered in patients with glossodynia, even those whose oral mucosa appears normal and who lack a history of gastrectomy.

## Background

Glossodynia is one of the most common oral symptoms in elderly people. This symptom has various etiologies, including trauma, local infection, anemia, diabetes mellitus, nutritional deficiencies, and trigeminal neuropathy [1–3].

Vitamin B_12_ is one of important nutritional components that affect oral health. Individuals with decreased levels of vitamin B_12_ have been reported to exhibit various oral manifestations such as glossitis, glossodynia, recurrent ulcers, cheilitis, dysgeusia, lingual paresthesia, burning sensations, and pruritus [4–8]. Moreover, 64.3 % of vitamin B_12_ deficiency patients (9 of 14 patients) with oral signs and symptoms were non-anemic and normocytic, suggesting the importance of more detailed blood screening in this patient group [9].

Most patients with vitamin B_12_ deficiency encountered in dental clinics have a history of gastrectomy due to gastric cancer. These patients have difficulty in absorbing vitamin B_12_ because the source of intrinsic factor, a glycoprotein known to be involved in vitamin B_12_ absorption in the ileum, is partly or totally eliminated by gastrectomy [10, 11]. However, vitamin B_12_ deficiency has also been observed in elderly patients who have never undergone gastrectomy. It has been reported that certain diseases such as pernicious anemia [12, 13], gastritis [6, 14, 15] and thyroid diseases [16, 17], or some medications [18–21] are related to the absorption process of vitamin B_12_. In patients without a history of gastrectomy, oral manifestations of vitamin B_12_ deficiency could be affected by the related medical conditions and/or medications. Therefore, this difference in etiological factors could result in variations in oral changes according to the presence or absence of a gastrectomy history. However, there have been no reports which compare oral symptoms of vitamin B_12_ deficiency patients with a history of gastrectomy with those without a history of gastrectomy.

In this study, we compared the clinical features of patients with vitamin B_12_ deficiency according to the presence or absence of a gastrectomy history. Probable etiologies of vitamin B_12_ deficiency in patients without a history of gastrectomy were also suggested.

## Methods

### Subjects

This study was a retrospective study based on chart review. Inclusion criteria was low vitamin B_12_ level (<200 pg/mL) and there was no specific exclusion criteria. Among the patients who were examined and treated by one doctor (HSK) in the Department of Oral Medicine at Seoul National University Dental Hospital (SNUDH) from January 2006 to January 2015, 22 patients were found to have a decreased level of vitamin B_12_ and were included in this study.

### Ethics

This chart review study was approved by the Institutional Review Board (IRB) of SNUDH (#CRI15013). The IRB authorized the exemption of informed consent from the subjects.

### Procedures

The oral symptoms, oral manifestations, blood examination results, and past medical history of each patient were reviewed. In addition, treatments, progression of oral symptoms, and medical consultation results were reviewed. Blood examinations were done during the initial evaluation before the commencement of treatments for oral symptoms. Among the results of blood examination, red blood cell (RBC) count (normal range: male, 4.2-6.3 x 10^6^/μL; female, 4.0-5.4 x 10^6^/μL), hemoglobin (Hb, normal range: male, 13-17 g/dL; female, 12-16 g/dL), hematocrit (Hct, normal range: male, 39-52 %; female, 36-48 %), mean corpuscular volume (MCV, normal range: male, 81-96 fL; female, 79-95 fL), mean corpuscular hemoglobin (MCH, normal range: male, 27-33 pg; female, 26-32 pg), mean corpuscular hemoglobin concentration (MCHC, normal range: 32-36 g/dL), vitamin B_12_ (normal range: 200-1000 pg/mL), folate (normal range: 3-15 ng/mL), and ferritin (normal range: 10-300 ng/mL) were included. When the result of vitamin B_12_ level was ‘<25 pg/mL’, the value of 25 pg/mL was used for the calculation of mean.

### Statistics

The significance of differences between the two groups was assessed by the Mann–Whitney U test (for continuous variables) and Fisher’s exact test (for categorical variables). For each test, a *P* value less than 0.05 was considered statistically significant.

## Results

The demographic characteristics of the patients with vitamin B_12_ deficiency are shown in Table 1. Of the total 22 patients, 11 had a history of gastrectomy (5 men and 6 women) and 11 did not (4 men and 7 women). Of 11 patients with a history of gastrectomy, 10 patients underwent gastrectomy due to gastric cancer and 1 patient due to abdominal rupture caused by a traffic accident. The two groups were not significantly different with respect to age (*P* = 0.323), duration of oral symptoms (*P* = 0.554), and vitamin B_12_ level (*P* = 0.895).

**Table 1:** Demographic characteristics of the patients with vitamin B_12_ deficiency

	With a history of gastrectomy	Without a history of gastrectomy	*P* value
	*N* = 11	*N* = 11	
Sex (male/female)	5/6	4/7	1.000
Age (years) Mean ± SD (range)	62.6 ± 7.6 (51–73)	66.9 ± 9.9 (52–85)	0.323
Symptom duration (months) Mean ± SD (range)	64.5 ± 61.7 (1–180)	37.6 ± 31.9 (6–96)	0.554
Vitamin B_12_ (pg/mL) Mean ± SD (range)	74.5 ± 51.2 (25–188)	64.2 ± 26.9 (25–123)	0.895

**Fig. 1:**
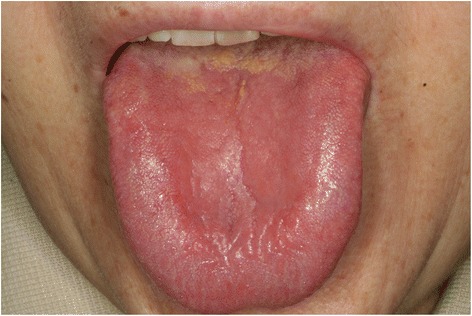
Image of the tongue in a patient with a history of gastrectomy (No. 4). Erythema and depapillation of the tongue were observed

**Fig. 2:**
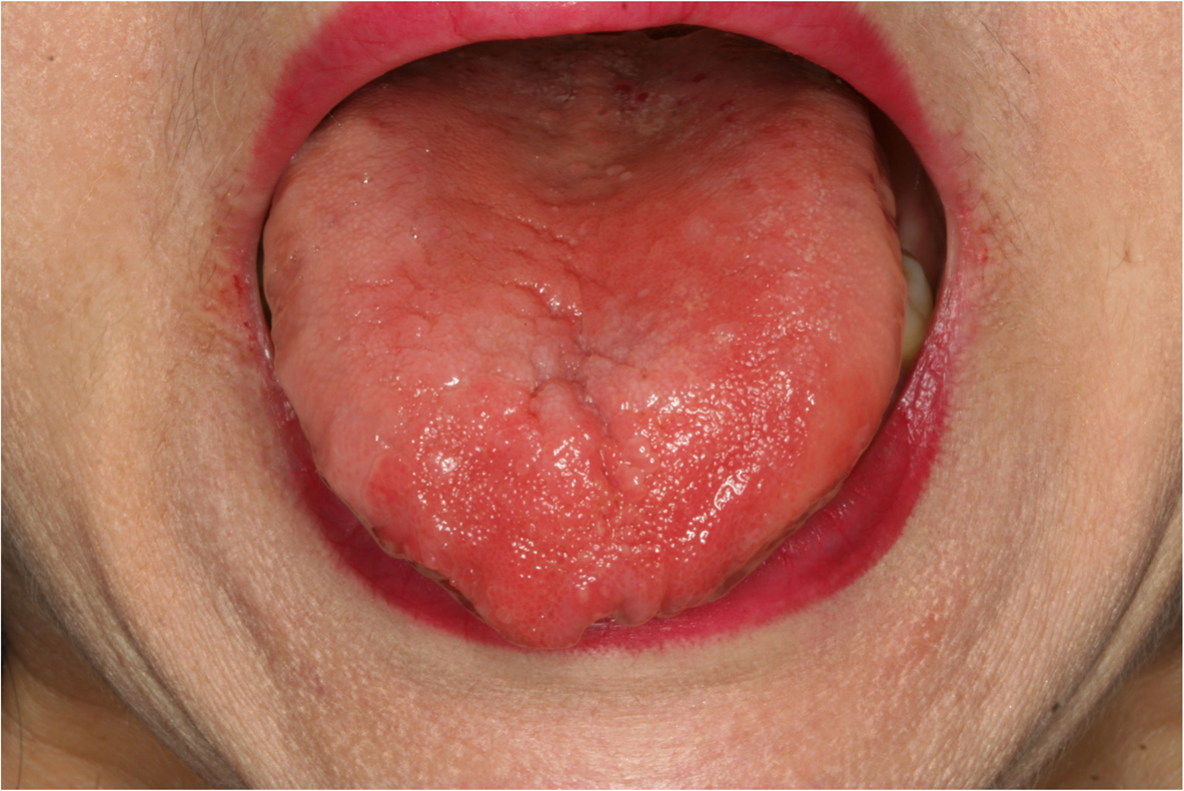
Image of the tongue in a patient without a history of gastrectomy (No. 3). Erythema and depapillation of the tongue were observed

**Fig. 3:**
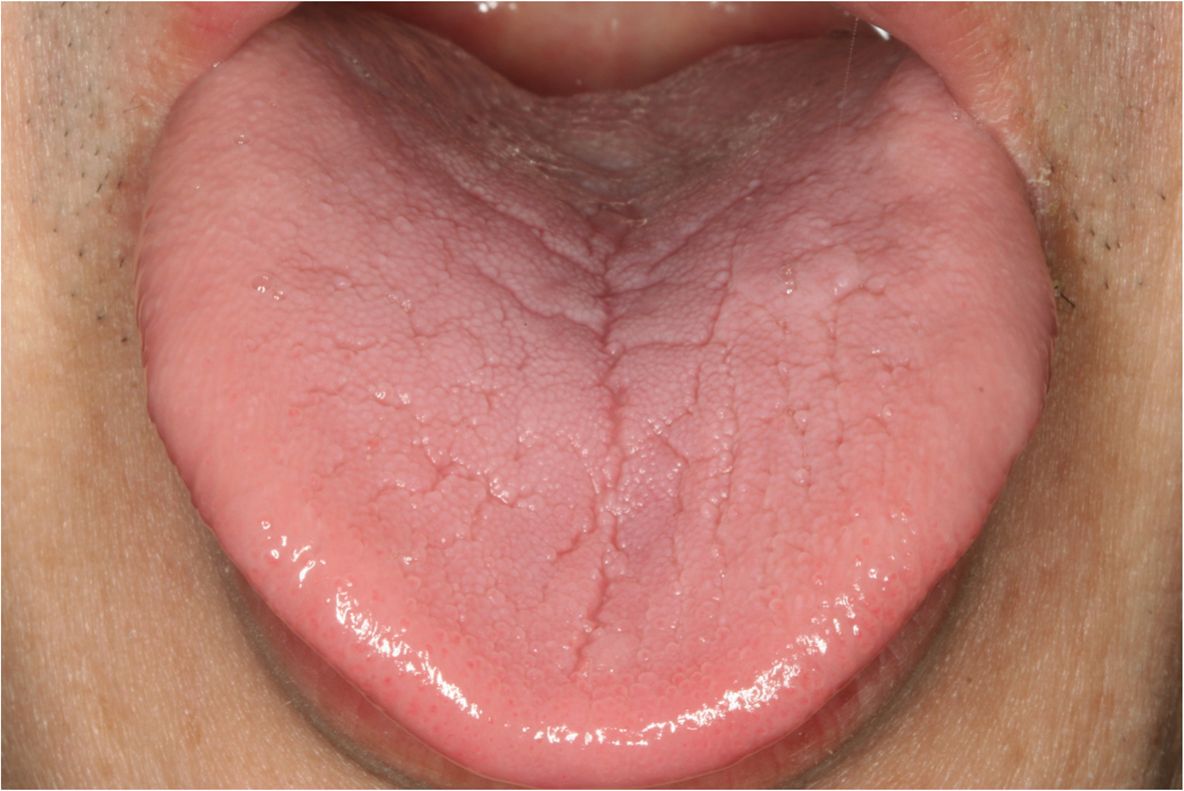
Image of the tongue in a patient with a history of gastrectomy (No. 5). He had suffered from tongue pain for 15 years, but no pathologic signs were observed on the tongue, except for tongue fissures

**Fig. 4:**
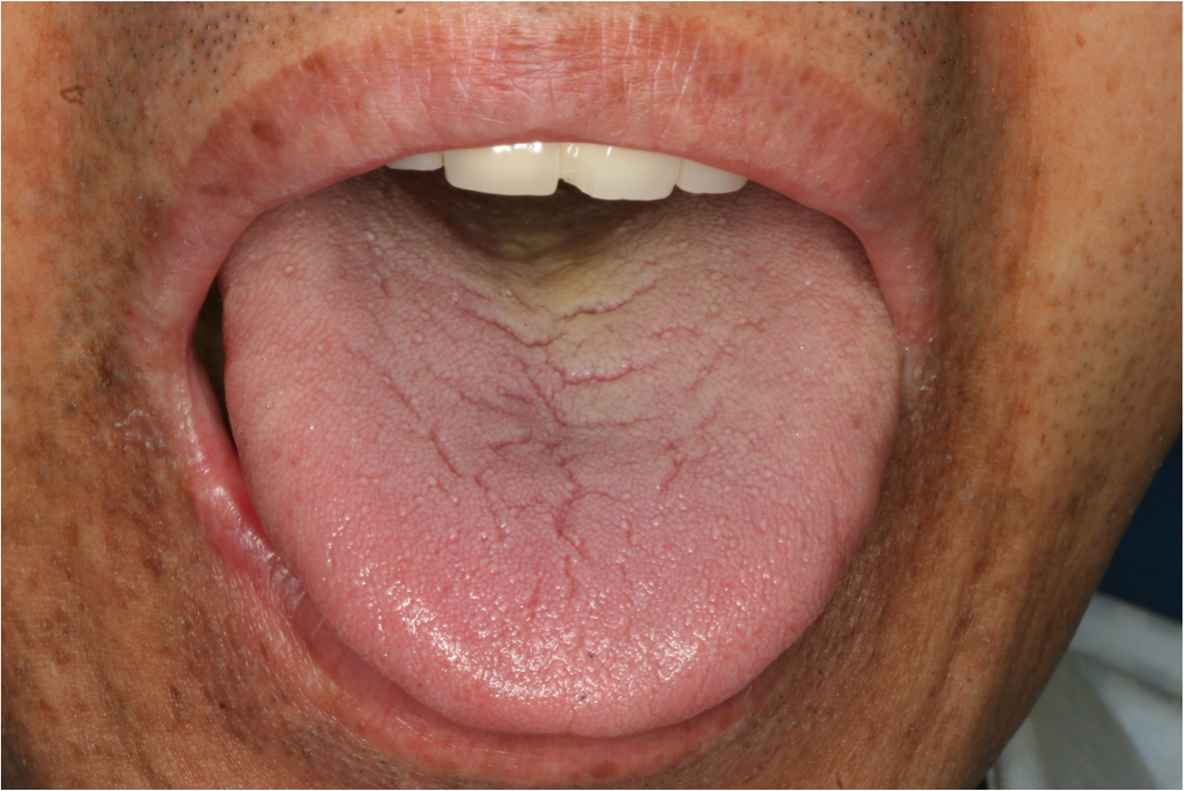
Image of the tongue in a patient without a history of gastrectomy (No. 4). No pathologic signs were observed on the tongue, except for tongue fissures and slight tongue coatings

The oral symptoms and findings from clinical examinations of the patients are shown in Table 2. The chief complaint was tongue pain for all patients. Other symptoms of the patients with a history of gastrectomy included dry mouth (6/11, 54.5 %) and pain in other intraoral mucosal areas (5/11, 45.5 %). The patients without a history of gastrectomy complained of pain in other intraoral mucosal areas (5/11, 45.5 %), dry mouth (3/11, 27.3 %), and dysgeusia (2/11, 18.2 %). Most of the patients from both groups were taking medications which could have been the cause for dry mouth. In the group with a history of gastrectomy, 1 patient was taking hypnotics and anti-parkinsonism drugs, 1 patient had a history of chemotherapy, and 1 patient was taking hypnotics and had a history of chemotherapy. Such medications and treatment history might be related with the increased incidence of dry mouth in the gastrectomy group. Erythema and depapillation of the tongue were the most common findings (Figs. 1 and 2). Patients with erythema of the tongue also had depapillation of the tongue. The patients with a history of gastrectomy showed such oral manifestations more frequently compared with those without a history of gastrectomy. Erythema and depapillation of the tongue were observed in 9 (81.8 %) patients with a history of gastrectomy and 6 (54.5 %) patients without a history of gastrectomy (*P* = 0.361). Angular cheilitis was present in 2 patients with a history of gastrectomy and 1 patient without a history of gastrectomy. Fissured tongue was observed in 8 patients of each group. Two patients (18.2 %) with a history of gastrectomy and 5 patients (45.5 %) without a history of gastrectomy had normal oral mucosa without erythema and depapillation of the tongue, or angular cheilitis (*P* = 0.361) (Figs. 3 and 4). Additionally, in the group with a history of gastrectomy, 1 patient showed erythema with erosion on the upper labial mucosa which seemed to be of a traumatic origin on the initial evaluation. The lesion was completely healed at the following appointment. Another patient showed whitish lichenoid lesions with erythema and erosion on both buccal mucosae. One patient without a history of gastrectomy showed erythema on both buccal mucosae which disappeared after antifungal therapy, suggesting the possibility of atrophic candidiasis.

**Table 2:** Oral symptoms and clinical findings in the patients with and without a history of gastrectomy

No	Age (years)	Sex	Chief complaint	Symptom duration (months)	Dry mouth	Pain in other oral mucosal areas	Dysgeusia	ET	DT	AC	FT	Other findings
With a history of gastrectomy												
1	68	F	Tongue pain	12	+	-	-	+	+	-	+	-
2	66	M	Tongue pain	1	-	+	-	+	+	-	+	erythema with erosion on the upper labial mucosa
3	59	F	Tongue pain	48	-	-	-	+	+	-	+	-
4	73	F	Tongue pain	14	+	-	-	+	+	-	+	-
5	53	M	Tongue pain	180	-	+	-	-	-	-	+	-
6	55	M	Tongue pain	60	-	+	-	-	-	-	-	whitish lesions with erythema and erosion on both buccal mucosa
7	51	F	Tongue pain	156	-	-	-	+	+	-	+	-
8	65	M	Tongue pain	12	+	-	-	+	+	-	-	-
9	57	F	Tongue pain	28	+	+	-	+	+	-	-	-
10	69	M	Tongue pain	54	+	+	-	+	+	+	+	-
11	73	F	Tongue pain	144	+	-	-	+	+	+	+	-
Without a history of gastrectomy												
1	63	F	Tongue pain	84	+	-	+	+	+	-	+	-
2	67	F	Tongue pain	72	-	-	-	+	+	-	-	-
3	73	F	Tongue pain	24	-	-	-	+	+	-	+	-
4	85	M	Tongue pain	12	-	+	-	-	-	-	+	-
5	56	F	Tongue pain	16	-	-	-	-	-	-	-	-
6	80	F	Tongue pain	6	+	-	-	-	-	-	+	-
7	60	F	Tongue pain	96	-	+	-	+	+	-	+	erythema on both buccal mucosa
8	70	F	Tongue pain	18	-	+	-	-	-	-	-	-
9	57	M	Tongue pain	18	-	+	-	+	+	-	+	-
10	73	M	Tongue pain	60	-	+	+	+	+	+	+	-
11	52	M	Tongue pain	8	+	-	-	-	-	-	+	-

ET, Erythema of the tongue; DT, Depapillation of the tongue; AC, Angular cheilitis; FT, Fissured tongue

The blood examination results of the patients are shown in Table 3. Although there were no significant differences in the mean values of blood examination results between the two groups (*P* = 0.081 - 0.974), it was notable that the patients with a history of gastrectomy tended to be more anemic. The RBC count was decreased in 8 patients (72.7 %) with a history of gastrectomy and 5 patients (45.5 %) without a history of gastrectomy (*P* = 0.387). The Hb level was decreased in 8 patients (72.7 %) with a history of gastrectomy and 3 patients (27.3 %) without a history of gastrectomy (*P* = 0.086). The Hct level was decreased in 7 patients (63.6 %) with a history of gastrectomy and 4 patients (36.4 %) without a history of gastrectomy (*P* = 0.395). The MCV was increased in 6 patients (54.5 %) in each group.

**Table 3:** Blood examination results of the patients with and without a history of gastrectomy

No	Age	Sex	Symptom duration	RBC	Hb	Hct	MCV	MCH	MCHC	Vit. B_12_	Folate	Ferritin
	(years)		(months)	(x10^6^/μL)	(g/dL)	(%)	(fL)	(pg)	(g/dL)	(pg/mL)	(ng/mL)	(ng/mL)
				M: 4.2-6.3	13-17	39-52	81-96	27-33	32-36	200-1000	3-15	10-300
				F: 4.0-5.4	12-16	36-48	79-95	26-32				
With a history of gastrectomy												
1	68	F	12	4.1	11.9	38.5	93.2	28.8	30.9	31.0	9.3	8.7
2	66	M	1	3.0	10.4	30.8	102.7	34.7	33.8	47.0	7.4	37.3
3	59	F	48	3.7	12.5	37.4	102.5	34.2	33.4	88.0	26.0	26.8
4	73	F	14	3.6	11.0	33.9	94.7	30.7	32.4	147.0	6.6	26.8
5	53	M	180	3.6	12.5	35.7	98.1	34.3	35.0	94.0	6.0	36.2
6	55	M	60	4.6	14.8	41.9	91.3	32.2	35.3	188.0	1.4	69.4
7	51	F	156	3.3	11.5	34.3	105.0	35.0	33.4	82.0	13.0	-
8	65	M	12	3.7	13.8	38.6	106.0	37.9	35.7	<25	14.1	30.2
9	57	F	28	3.6	8.9	29.8	84.0	25.2	30.0	68.0	17.5	3.0
10	69	M	54	2.6	11.4	34.3	130.0	43.1	33.2	25.0	75.0	264.6
11	73	F	144	4.2	11.8	36.7	86.9	28.1	32.3	<25	14.0	-
Mean	62.6		64.5	3.6	11.9	35.6	99.5	33.1	33.2	85.6	17.3	55.9
Without a history of gastrectomy												
1	63	F	84	3.9	12.0	35.9	93.0	31.0	33.4	<25	14.4	45.4
2	67	F	72	4.6	13.2	38.8	83.8	28.5	34.0	123.0	7.1	47.8
3	73	F	24	4.8	14.4	41.7	87.2	30.2	34.6	62.0	30.0	17.7
4	85	M	12	2.9	11.0	31.6	109.0	38.0	34.7	56.0	8.0	144.0
5	56	F	16	4.9	13.5	40.0	81.5	27.5	33.7	107.0	1.5	39.7
6	80	F	6	3.9	13.8	38.9	100.0	35.6	35.6	67.0	15.3	-
7	60	F	96	3.7	11.0	35.4	96.8	30.1	31.1	47.0	18.4	7.5
8	70	F	18	4.6	15.3	44.5	96.1	33.0	34.4	43.0	0.3	167.2
9	57	M	18	1.5	6.5	20.2	132.0	42.7	32.4	47.0	7.6	424.1
10	73	M	60	4.4	14.7	42.0	96.0	33.7	35.1	60.0	17.9	181.5
11	52	M	8	4.3	15.5	44.4	103.0	36.0	34.9	69.0	19.8	27.4
Mean	66.9		37.6	4.0	12.8	37.6	98.0	33.3	34.0	68.1	12.8	110.2

RBC, red blood cell count; Hb, hemoglobin; Hct, hematocrit; MCV, mean corpuscular volume; MCH, mean corpuscular hemoglobin; MCHC, mean corpuscular hemoglobin concentration; Vit., vitamin

When the result of vitamin B_12_ level was ‘<25 pg/mL’, the value of 25 pg/mL was used for the calculation of mean

No significant differences were found between the blood examination results of the patients with and without a history of gastrectomy (the Mann–Whitney U test, *P* > 0.05)

For management of oral symptoms, topical antifungal therapies were administered to 9 patients with a history of gastrectomy. These therapies included nystatin suspension (4 mL of 100,000 units/mL, 3 times/day, topical) for 5 patients, nystatin suspension with clonazepam (0.5 mg/day, topical) for 1 patient, fluconazole suspension (2.5 mL of 10 mg/mL, 2 times/day, topical) for 1 patient, fluconazole suspension with clonazepam (0.5 mg/day, topical) for 1 patient, and both nystatin and itraconazole suspensions (5 mL of 10 mg/mL, 2 times/day, topical) for 1 patient. One patient was prescribed carboxymethylcellulose (CMC)-based artificial saliva only, and 1 patient was referred to a physician at the first visit. Of the 9 patients who received topical antifungal therapy, 8 showed symptom improvement. All patients with a history of gastrectomy were referred to physicians for further evaluation and management. The results of only 9 patients were available, because 2 patients did not visit after the referrals. Intramuscular injection of vitamin B_12_ was performed in 4 patients and the oral symptoms improved in all. Oral replacement therapy of vitamin B_12_ was performed in 3 patients. The symptoms disappeared in 2 of these patients and partially improved in 1 of these patients. One patient received both vitamin B_12_ injection and oral folate replacement therapy; these treatments led to symptom improvement. One patient received oral vitamin B_12_, ferritin, folate replacement therapy, and vitamin B_12_ injection and this therapy was effective.

Regarding the patients without a history of gastrectomy, topical antifungal therapy (nystatin suspension 4 mL of 100,000 units/mL, 3 times/day, topical) was administered to 6 patients, CMC-based artificial saliva and clonazepam (0.5 - 1.0 mg/day, topical and/or p.o.) to 4 patients, and CMC-based artificial saliva only to 1 patient. Of the 5 patients who did not undergo antifungal therapy, 4 patients did not have any pathologic oral signs, with the exception of fissured tongue. All 6 patients who received topical antifungal therapy and all 4 patients who received CMC-based artificial saliva and clonazepam showed symptom improvement. The one patient who received CMC-based artificial saliva only did not show symptom improvement. Of the 11 patients in this group, 9 were referred to physicians for further evaluation and management. One patient refused the referral after exhibiting significant symptom improvement. The other patient could not be referred because the patient did not come to the clinic on the day that the referral was scheduled. Of the 9 referred patients, 3 were treated with oral vitamin B_12_ replacement therapy, 2 were given vitamin B_12_ injection, and 2 were treated with both oral vitamin B_12_ replacement and injection. All of them showed symptom improvement. One patient was treated with oral vitamin B_12_ and iron replacement therapy; this treatment was effective. The other patient who underwent gastroscopy was diagnosed with early gastric cancer and underwent gastrectomy.

The probable etiologies of vitamin B_12_ deficiency in the patients without a history of gastrectomy are shown in Table 4. Five patients (No. 1, 3, 5, 6, and 8) suffered from gastritis, which might have caused the vitamin B_12_ deficiencies in these patients. Three patients (No. 1, 2, and 4) had diabetes mellitus and were taking related medications, indicating that their diabetes medications were the probable etiologies. One of these patients (No. 1) was also taking thyroid hormone after thyroidectomy due to thyroid cancer. One patient (No. 7) was vegetarian; thus, insufficient intake of nutritional components containing vitamin B_12_ might have caused the deficiency. An additional blood examination performed in the department of hematooncology revealed that one patient (No. 9) had antibodies against the intrinsic factor. Another patient (No. 10) was diagnosed with early gastric cancer based on the result of a gastroscopy performed in the department of internal medicine. One patient (No. 11) underwent gastroscopy, which yielded normal results. No probable etiologic factors were reported in any of the medical histories or additional examinations done at the clinic to which the patient was referred.

**Table 4:** Probable etiologies in the patients without a history of gastrectomy

No	Age (years)	Sex	Symptom duration (months)	Probable etiology of vitamin B_12_ deficiency
1	63	F	84	Medications for diabetes mellitus and Gastritis
2	67	F	72	Medications for diabetes mellitus
3	73	F	24	Gastritis
4	85	M	12	Medications for diabetes mellitus
5	56	F	16	Gastritis
6	80	F	6	Gastritis
7	60	F	96	Vegetarian diet
8	70	F	18	Gastritis
9	57	M	18	Antibodies to intrinsic factor
10	73	M	60	Early gastric cancer
11	52	M	8	Unknown

Among 11 patients with a history of gastrectomy, 2 patients (No. 3 and 8) had gastritis, 2 patients (No. 4 and 11) had diabetes mellitus and were taking related medications, and 1 patient (No. 1) was taking thyroid hormone for hypothyroidism.

## Discussion

Our results showed that oral signs and symptoms and blood examination abnormalities were more common and also more severe in patients with a history of gastrectomy. Resection of the gastrointestinal tract was the definite cause although some of these patients had other medical conditions which might have played a role in the pathogenesis of the vitamin B_12_ deficiency. On the other hand, in patients without a history of gastrectomy, the severity and duration of diseases and/or the dose and duration of medication intake could have affected the clinical and laboratory results. Interestingly, 2 patients with a history of gastrectomy and 5 patients without a history of gastrectomy did not show any significant pathologic oral signs, except for tongue fissures. Decreased Hb and Hct levels were more common in patients with a history of gastrectomy compared to those without a history of gastrectomy. The probable etiologies for the vitamin B_12_ deficiencies in the patients without a history of gastrectomy were gastritis, diabetes medications, a vegetarian diet, antibodies to the intrinsic factor, and early gastric cancer.

Atrophic gastritis, a very common disease with a high prevalence in elderly patients, has been known to be one of the most common causes of vitamin B_12_ deficiency [6, 14, 15]. Chronic inflammation of the stomach wall causes atrophy of the gastric mucosa and decreased secretion of gastric acid, which can result in malabsorption of vitamin B_12_. Moreover, medications for gastritis, such as proton pump inhibitors, have been reported to inhibit gastric acid production, which might also cause malabsorption of vitamin B_12_ [18, 20].

Metformin is one of the most commonly prescribed drugs for type 2 diabetes and is well known to be associated with vitamin B_12_ deficiency [19, 21, 22]. Metformin inhibits gluconeogenesis, decreases hepatic glucose output, and increases insulin sensitivity. One of the most commonly reported side effects of metformin is gastrointestinal disorders, including reduced vitamin B_12_ absorption. Metformin disturbs the metabolism of calcium which is one of the necessary elements for the body to absorb vitamin B_12_ [22].

Although pernicious anemia is uncommon in Asians including Korean ethnicity [23, 24], this disease is another cause of vitamin B_12_ deficiency [12, 13]. Pernicious anemia is an autoimmune disease characterized by the absence of intrinsic factor, a glycoprotein that is necessary for vitamin B_12_ absorption [25, 26]. This condition prevents the normal absorption of vitamin B_12_, thereby resulting in vitamin B_12_ deficiency. Vitamin B_12_ is usually found in foods of animal origin, such as meat, poultry, fish, and eggs [7]. Therefore, a strict vegetarian diet could cause a vitamin B_12_ deficiency, and vitamin B_12_ replacement is recommended for vegetarians. Thyroid diseases have also been known to be associated with vitamin B_12_ deficiency [16, 17]. Thyroid hormone stimulates erythropoiesis and anemia frequently develops in patients with thyroid hormone disorders. Megaloblastic anemia has been reported to be related to thyroid diseases, but this relationship is still controversial [27, 28].

The finding that 5 of 11 vitamin B_12_ deficiency patients without a history of gastrectomy complained of tongue pain in the absence of any significant pathologic oral signs suggests that blood examinations, including vitamin B_12_ measurements, are mandatory for patients with glossodynia. Such examinations are important even for patients without a history of gastrectomy and for patients without any pathologic oral signs. Furthermore, the finding that 1 patient had early gastric cancer implies that gastroscopy is necessary for patients who have not undergone gastroscopy regularly.

Antifungal therapy was effective especially in patients with oral signs such as tongue erythema and depapillation, or angular cheilitis. Since vitamin B_12_ deficiency can cause an anemic state in the body by attenuating the immune system, patients with vitamin B_12_ deficiency are more susceptible to opportunistic infections such as candidiasis [29]. Some patients treated with clonazepam exhibited symptom improvement. Clonazepam is the preferred drug for treating burning mouth syndrome and has been widely used as a topical agent, an oral agent, and a combined way [30–33]. Vitamin B_12_ deficiency has been reported to be related to peripheral neuropathy [7, 34]. Thus, some of the oral symptoms in our patients may be related to neuropathic changes of the trigeminal nerve.

As expected, vitamin B_12_ replacement therapy was effective for most patients, regardless of their gastrectomy history. Interestingly, oral vitamin B_12_ replacement therapy was also effective for the patients with a history of gastrectomy. Orally taken vitamin B_12_ can be absorbed by an intrinsic factor-independent passive diffusion pathway. Oral vitamin B_12_ replacement has been reported to be effective and safe treatment, even in patients with a history of total gastrectomy [10].

Our study showed that the most common oral symptom in patients with vitamin B_12_ deficiency was tongue pain and the most common findings were erythema and depapillation of the tongue. These oral signs and symptoms and blood examination abnormalities were less common and also less severe in patients without a history of gastrectomy than those with a history of gastrectomy. Oral symptoms responded to antifungal therapy. Clonazepam could be of additional help. Vitamin B_12_ replacement therapy was effective. Patients without a history of gastrectomy exhibited many probable etiologic factors, such as gastritis, medications for diabetes and/or gastritis, a vegetarian diet, autoimmunity, and gastric cancer.

## Conclusions

It is essential that complete medical histories including medication information should be obtained from all patients complaining of tongue pain, irrespective of their oral findings or gastrectomy history. Gastroscopy is strongly recommended for all patients with vitamin B_12_ deficiency who do not have a history of gastrectomy.

## Abbreviations

CMC, carboxymethylcellulose; Hb, Hemoglobin; Hct, Hematocrit; MCV, mean corpuscular volume; RBC, red blood cell
